# Prognosis of hemodialysis patients with progressive aortic stenosis: a prospective cohort study

**DOI:** 10.1186/s41100-021-00367-3

**Published:** 2021-09-08

**Authors:** Yoriko Horiguchi, Kaoru Uemura, Naoyoshi Aoyama, Shinichi Nakajima, Tomoki Asai, Sachiko Motohashi, Manae Harada, Maoko Ida, Atsushi Yoshida

**Affiliations:** 1 Department of Cardiology, Sagami Circulatory Organ Clinic, 4-21-15 Sounan, Minami-ku, Sagamihara, Kanagawa 252-0312 Japan; 2 Department of Clinical Laboratory, Sagami Circulatory Organ Clinic, Sagamihara, Kanagawa Japan; 3 Department of General Medicine, Kitasato University School of Medicine, Sagamihara, Kanagawa Japan; 4 Department of Internal Medicine, Sagami Circulatory Organ Clinic, Sagamihara, Kanagawa Japan; 5 Department of Rehabilitation, Sagami Circulatory Organ Clinic, Sagamihara, Kanagawa Japan

**Keywords:** Aortic stenosis, Cardiac death, Cardiac events, Hemodialysis, Mortality

## Abstract

**Background:**

Whether progressive mild to moderate aortic stenosis in hemodialysis patients influences their prognosis has not been elucidated. This prospective cohort study explored whether progressive aortic stenosis predicted the rate of cardiac events and mortality in those patients.

**Methods:**

A total of 283 consecutive hemodialysis patients (no aortic stenosis, 248; progressive aortic stenosis, 35) underwent echocardiography for assessment of aortic stenosis, with a median follow-up period of 4.1 years. Study endpoints were cardiac events, all-cause mortality, and cardiac death. Kaplan–Meier analysis and multivariate Cox proportional hazard analysis were performed to estimate cardiac events, all-cause mortality, and cardiac death.

**Results:**

Cumulative cardiac event rate, all-cause mortality rate, and the rate of cardiac death at 3-year follow-up were 44.9%, 40.5%, and 26.4% in patients with progressive aortic stenosis and 22.1%, 19.0%, and 7.5% in those without aortic stenosis, respectively. Kaplan–Meier analysis demonstrated the cumulative rates of cardiac events and all-cause mortality. And cardiac death was significantly higher in patients with progressive aortic stenosis than in those without aortic stenosis. Multivariate Cox proportional hazard analysis revealed that progressive aortic stenosis was predictive of cardiac events (adjusted hazard ratio 2.47; 95% confidence interval 1.38–4.39) and cardiac death (adjusted hazard ratio 4.21; 95% confidence interval 2.10–8.46). Age, physical activity, C-reactive protein, and serum albumin levels—but not progressive aortic stenosis—predicted all-cause mortality.

**Conclusions:**

The rates of cardiac events and cardiac death were higher in hemodialysis patients with progressive aortic stenosis than in those without aortic stenosis. Furthermore, progressive aortic stenosis predicted cardiac events and cardiac death. Compared with those without aortic stenosis, patients with progressive aortic stenosis had higher all-cause mortality, which was related to their comorbidities.

*Trial registration* This study was retrospectively registered with University Hospital Medical Information Network Clinical Trials Registry (registration number, UMIN 000024023) at September 12th, 2016.

## Introduction

Patients on hemodialysis often develop aortic stenosis (AS) after aortic valve calcification [1, 2]. Severe AS is present in 4 to 13% of patients undergoing hemodialysis and is correlated with their poor survival [1, 3, 4]. In the general population, patients with progressive mild to moderate AS have a higher rate of cardiovascular events than those without AS, and moderate AS is associated with increased all-cause mortality [5–8]. Because of its association with cardiac valvular calcification, which influences the prognosis in hemodialysis patients, progressive AS may also influence the prognosis in hemodialysis patients. Knowing the prognosis of patients with progressive AS is crucial to managing them appropriately [3, 9]. Whether progressive AS in hemodialysis patients influences their rate of cardiac events and mortality has not been elucidated. The aim of this prospective cohort study was to explore the prognostic influence of progressive AS on morbidity related to cardiac events and mortality in hemodialysis patients.

## Materials and methods

### Study population and echocardiography

This single-center, prospective cohort study was conducted in accordance with the ethical principles for medical research involving human subjects as delineated in the Declaration of Helsinki. This study was approved by the institutional review board of our institution, and written informed consent was obtained from each participant.

A total of 375 consecutive patients with end-stage renal disease who underwent maintenance hemodialysis at our institution from February 2016 through November 2017 were enrolled in our study. We excluded 78 patients who did not give informed consent and 8 patients who had undergone aortic valve replacement. Therefore, 289 patients underwent transthoracic echocardiography in this study.

We performed echocardiography of the patients by using an Aplio a Verifia system (Canon Medical Systems Corporation, Tochigi, Japan) according to the guidelines released by the American Society of Echocardiography and the European Association of Cardiovascular Imaging [10, 11]. Left ventricular volume and ejection fraction were calculated by using the biplane disk summation method [10]. We measured the aortic valve area (AVA) in each patient with AS, which was calculated by using the continuity equation, peak aortic jet velocity (aortic *V*_max_), mean pressure gradient across the aortic valve (mean ∆*P*), and stroke volume index (SVI) [11]. We classified the severity of AS according to the 2014 American Heart Association/American College of Cardiology guidelines for the management of patients with valvular heart disease as follows: no AS, aortic *V*_max_ < 2 m/s; progressive AS, AVA > 1.0 cm^2^ or AVA ≤ 1.0 cm^2^ with SVI ≥ 35 mL/m^2^, aortic *V*_max_ of 2.0–3.9 m/s, or mean ∆*P* < 40 mmHg; severe AS (high gradient), AVA ≤ 1.0 cm^2^, aortic *V*_max_ ≥ 4.0 m/s or mean ∆*P* ≥ 40 mmHg; and severe AS (low gradient), AVA ≤ 1.0 cm^2^ with SVI < 35 mL/m^2^, aortic *V*_max_ < 4.0 m/s, and mean ∆*P* < 40 mmHg [12]. Six patients were diagnosed to have severe AS and were therefore excluded from the study. Consequently, 283 patients (no AS, 248; progressive AS, 35) were evaluated in this study.

### Assessment of clinical status

We assessed participants’ physical activity by using the Specific Activity Scale (SAS) questionnaire within a week before or after the day on which echocardiography was performed [13]. The SAS score (metabolic equivalents [METs]) was determined as the lowest intensity of activity that the patients could not perform. We then asked the patients whether they had had exercise-related symptoms of dyspnea, chest pain, or syncope within the past 2 months.

Blood tests were performed within 2 weeks before or after the day when echocardiography was done. Blood samples were collected before the first hemodialysis procedure of the week. We examined the medical records of patients for history of admission on account of ischemic heart disease or heart failure.

Peripheral artery disease (PAD) was diagnosed in patients with abnormal ankle brachial index (i.e., ≤ 0.9) or toe brachial index (i.e., ≤ 0.6), in those with lower limb arterial echography showing > 50% stenosis, and those with a history of peripheral vascular revascularization or limb amputation due to PAD [14].

### Endpoints

Patient follow-up commenced on the day when echocardiography was performed and continued until January 2021. We included patients who were lost to follow-up in the analyses. We noted the timing of cardiac events and all-cause mortality in the study patients. Cardiac events included aortic valve replacement, hospital admission due to heart failure, acute coronary syndrome, coronary artery revascularization, resuscitated arrhythmia and cardiac arrest, and cardiac death during the follow-up period. Cardiac death was defined as death due to heart failure, acute myocardial infarction, or fatal arrhythmia or sudden death.

### Statistical analysis

The statistical power of the survival analysis at the two-sided significance level of 0.05 was 0.91 when the 35 patients with progressive AS had a 22% higher 3-year event rate than the 248 patients without AS.

Continuous values were expressed as means ± 1 standard deviation. To assess patient characteristics, we used the unpaired or paired *t*-test, one-way analysis of variance, Tukey test, and nonparametric tests (Mann–Whitney *U* test or Kruskal–Wallis test) for continuous variables and the Chi-square test or Fisher exact test for categorical variables. The rates of all-cause mortality, cardiac events, and cardiac death were analyzed by using the Kaplan–Meier method with log-rank testing. We used Cox proportional hazard analysis to assess which variables were related to all-cause mortality, cardiac events, and cardiac death. First, we conducted univariate Cox proportional hazard analysis. We analyzed the independent variables of age, male gender, body mass index, length of time on dialysis, SAS score, presence of progressive AS, PAD, history of diabetes, hypertension, dyslipidemia, ischemic heart disease, heart failure, cerebrovascular disease, present tobacco use, hemoglobin, serum level of albumin, adjusted calcium, phosphorus, calcium–phosphorus product, C-reactive protein, and low-density lipoprotein. Then, we performed multivariate Cox proportional hazard analysis adjusted for statistically significant independent variables (i.e., *P* < 0.05) in the univariate Cox proportional hazard analysis. To evaluate prognostic factors affecting the onset of cardiac death, we performed stepwise multivariate Cox proportional hazard analysis.

A *P* value of less than 0.05 was considered to indicate statistical significance. All statistical analyses were performed by using IBM SPSS Statistics (version 22, IBM Corporation, Armonk, NY, USA) and EZR version 1.41 (R version 3.6.1) [15].

## Results

### Patients

Study participants (*n* = 283) were followed for a median period of 4.1 years (interquartile range 2.8–4.4 years). Fifteen (5.3%) patients were lost to follow-up during the study period. A total of 190 (96.0%) and 144 (72.7%) of the 198 surviving patients had a minimum follow-up period of 3 and 4 years, respectively.

The baseline characteristics of the study patients are shown in Table 1. No patient had bicuspid aortic valve. Patients with progressive AS were significantly older and had lower SAS scores and higher prevalence of PAD than those without AS (Table 1). The frequency of the exercise-induced symptoms did not differ significantly between these patient groups (Table 1).

**Table 1:** Baseline characteristics of the study patients

Parameter	Patients with progressive AS	Patients without AS	*P*
Number of patients	35	248	
Age (year)	74 ± 11	67 ± 12	< 0.001
Male gender, *n* (%)	20 (57)	144 (58)	1.00
Body mass index (kg/m^2^)	21.0 ± 4.0	21.9 ± 4.6	0.27
Length of time on dialysis (year)	12 ± 9	9 ± 9	0.08
Specific Activity Scale (METs)	3.5 ± 2.0	4.3 ± 2.1	0.03
Exercise-related symptoms, *n* (%)			
Syncope	5 (14)	16 (7)	0.16
Chest pain	7 (20)	54 (22)	1.00
Shortness of breath	12 (34)	65 (26)	0.32
Symptom, any of the above	12 (34)	98 (40)	0.59
Coexisting conditions, *n* (%)			
Diabetes	13 (37)	107 (43)	0.59
Hypertension	30 (86)	192 (77)	0.38
Dyslipidemia	14 (40)	101 (41)	1.00
Ischemic heart disease	9 (26)	60 (24)	0.84
Heart failure	11 (31)	45 (18)	0.07
Atrial fibrillation	3 (8)	26 (11)	1.00
Peripheral artery disease	20 (57)	75 (30)	0.004
History of PTA or limb amputation	8 (23)	34 (14)	0.20
Cerebrovascular disease	9 (26)	51 (21)	0.51
Past or present tobacco use	15 (43)	102 (41)	0.86
Medications, *n* (%)			
ACE inhibitor	1 (3)	9(4)	1.00
ARB	14 (40)	93 (38)	0.85
Calcium channel blocker	22 (63)	132 (53)	0.37
Beta blocker	10 (29)	91 (37)	0.45
Statin	14 (40)	101 (41)	1.00
Vitamin D_3_	14 (40)	127 (51)	0.28
Phosphate binder	13 (37)	88 (35)	0.85
Calcimimetics	5 (14)	29 (12)	0.59
Calcium carbonate	26 (74)	160 (65)	0.34
Blood test results			
Hemoglobin (g/dL)	10.7 ± 1.3	10.8 ± 1.1	0.72
Albumin (g/dL)	3.5 ± 0.4	3.6 ± 0.4	0.08
Adjusted calcium (mg/dL)	9.2 ± 0.6	9.0 ± 0.8	0.12
Phosphorus (mg/dL)	5.1 ± 1.4	5.4 ± 1.3	0.27
Calcium–phosphorus product (mg^2^/dL^2^)	47.9 ± 12.7	47.2 ± 13.2	0.78
C-reactive protein (mg/dL)	2.1 ± 5.4	0.6 ± 1.9	0.18
LDL cholesterol (mg/dL)	81 ± 24	81 ± 22	0.68

*ACE* angiotensin-converting enzyme, *ARB* angiotensin II receptor blocker, *AS* aortic stenosis, *LDL* low-density lipoprotein, *METs* metabolic equivalents, *PTA* percutaneous transluminal angioplasty

Echocardiographic data showed that the left ventricular volume and ejection fractions of patients with progressive AS were similar to those without AS (Table 2). The ratio between early mitral inflow velocity and mitral annular early diastolic velocity (*E*/*e*′) was measured in 165 patients (patients without AS, 146; progressive AS, 19). *E*/*e*′ was higher in those with progressive AS (17.0 ± 8.4) than those without AS (11.9 ± 4.6, *P* = 0.015).

**Table 2:** Echocardiographic data of the study patients

Parameter	Patients with progressive AS	Patients without AS	*P*
Number of patients	35	248	
LVEDVI (mL/m^2^)	105 ± 22	97 ± 31	0.12
LVESVI (mL/m^2^)	50 ± 20	45 ± 20	0.21
LVEF (%)	56 ± 13	56 ± 14	0.93
LADs (mm)	40 ± 7	39 ± 20	0.62
IVSd (mm)	10 ± 2	10 ± 3	0.96
PWd (mm)	10 ± 2	10 ± 2	0.98
*E*/*A*	0.85 ± 0.30	0.86 ± 0.93	0.89
AVA (cm^2^)	1.5 ± 0.4	NA	
Mean aortic PG (mmHg)	18.7 ± 17.8	NA	
Aortic *V*_max_ (cm/s)	269.4 ± 43.1	NA	

*AS* aortic stenosis, *AVA* aortic valve area, *E/A* the ratio of the early and late diastolic trans mitral flow velocity, *IVSd* interventricular septum thickness at end diastole, *LADs* left atrial dimension at end systole, *LVEDVI* left ventricular end-diastolic volume index, *LVEF* left ventricular ejection fraction, *LVESVI* left ventricular end-systolic volume index, *NA* not available, *PG* pressure gradient, *PWd* posterior left ventricular wall thickness at end diastole, *V*_*max*_ maximum velocity

### Follow-up and endpoints

During follow-up, 5 (1.8%) patients developed severe AS and 2 (0.7%) underwent aortic valve replacement. In addition, 1 (0.4%) patient developed coronavirus disease 2019 and recovered without cardiovascular complication. A total of 85 (30.0%) patients (with progressive AS, 18; without AS, 67) experienced cardiac events. These cardiac events included hospitalization for heart failure for 44 (15.5%) patients, coronary revascularization and acute coronary syndrome in 29 (10.2%), sudden death in 6 (2.1%), fatal or resuscitated arrhythmia in 4 (1.4%), and aortic valve intervention in 2 (0.7%). A total of 86 (30.4%) patients (with progressive AS, 19; without AS, 67) died due to various causes during follow-up (cardiac death, 38 [13.4%]; infection, 25 [8.8%]; malignancy, 7 [2.5%]; gastrointestinal bleeding, 2 [0.7%]; lethal hyperkalemia, 1 [0.4%]; stroke, 1 [0.4%]; extrinsic death, 2 [0.7%]; cause unknown, 10 [3.5%]). Cardiac death occurred in 12 patients with progressive AS and 26 in those without AS, respectively. The causes of the cardiac death were: heart failure in 17 (6.0%) patients, sudden death in 10 (3.5%), acute myocardial infarction in 8 (2.8%), and fatal arrhythmia in 3 (1.1%).

**Fig. 1:**
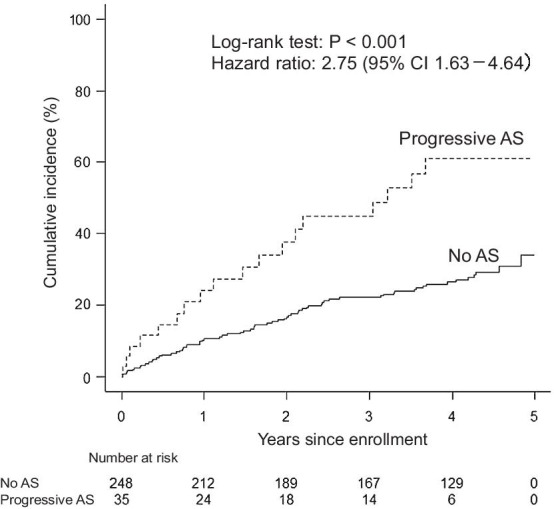
Kaplan–Meier curves related to cardiac events. The log-rank test revealed that the cardiac event rate at the entire follow-up was higher in patients without aortic stenosis (AS) than in those with progressive AS. Solid line, no AS; dashed line, progressive AS

The cumulative cardiac event rate at the 3-year follow-up was 44.9% in patients with progressive AS and 22.1% in those without AS (Fig. 1). Kaplan–Meier analysis revealed that the cumulative cardiac event rate was higher in patients with progressive AS than in those without AS (the 3-year follow-up*, P* = 0.002; the entire follow-up, *P* < 0.001) (Fig. 1). The patients with progressive AS had a higher cumulative rate of composite coronary events than those without AS (28.2% vs. 9.1% at the 3-year follow-up, *P* = 0.003; 69.0% vs. 23.3% at the end of follow-up, *P* = 0.004), but their cumulative rate of heart failure hospitalization was comparable to that of those without AS (22.4% vs. 13.3% at the 3-year follow-up, *P* = 0.105; 33.5% vs. 27.4% at the final follow-up, *P* = 0.120).

**Fig. 2:**
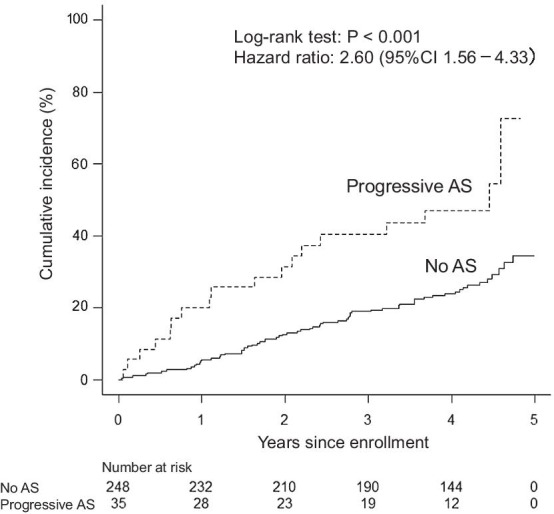
Kaplan–Meier curves related to all-cause mortality. The log-rank test showed that all-cause mortality at the entire follow-up was higher in patients without aortic stenosis (AS) than in those with progressive AS. Solid line, no AS; dashed line, progressive AS

**Fig. 3:**
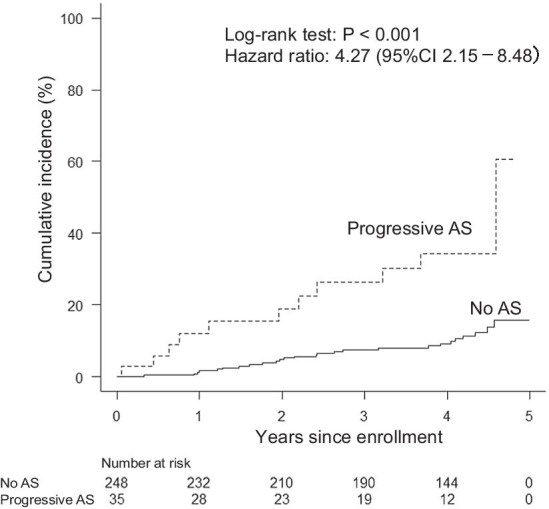
Kaplan–Meier curves related to cardiac death. The log-rank test demonstrated that cardiac death throughout follow-up was higher in patients without aortic stenosis (AS) than in those with progressive AS. Solid line, no AS; dashed line, progressive AS

The cumulative incidence of all-cause mortality at the 3-year follow-up was 40.5% in patients with progressive AS and 19.0% in those without AS. Similarly, Kaplan–Meier analysis demonstrated that the cumulative rate of all-cause mortality was higher in the patients with progressive AS than in those without AS (the 3-year follow-up*, P* < 0.001; the entire follow-up, *P* < 0.001) (Fig. 2). The cumulative rate of cardiac death at the 3-year follow-up was 26.4% in patients with progressive AS and 7.5% in those without AS. Kaplan–Meier analysis revealed a higher cumulative rate of cardiac death in patients with progressive AS than in those without AS (the 3-year follow-up*, P* < 0.001; the entire follow-up, *P* < 0.001) (Fig. 3). Throughout follow-up among the patients with progressive AS, cardiac death occurred in 6 of the 9 patients with a history of ischemic heart disease, in 4 of the 11 patients with a history of heart failure, and in 3 of the 8 patients with no history of ischemic heart disease or heart failure.

Univariate Cox proportional hazard analysis identified the following independent variables as predictors of cardiac events: age, male gender, body mass index, SAS score, progressive AS, history of diabetes, ischemic heart disease, heart failure, and PAD. Multivariate Cox proportional hazard analysis revealed that independent predictors of cardiac events were history of ischemic heart disease (adjusted hazard ratio 2.50; 95% confidence interval [CI] 1.55–4.03; *P* < 0.001) and presence of progressive AS (adjusted hazard ratio 2.47; 95% CI 1.38–4.39; *P* = 0.002) (Table 3).

**Table 3:** Multivariate Cox proportional hazard analysis regarding prediction of cardiac events

Variable	Adjusted hazard ratio	95% Cl	*P*
Age (per 1 year)	1.02	1.00–1.04	0.13
Male gender	1.38	0.84–2.26	0.20
Body mass index (per 1 kg/m^2^)	1.05	0.99–1.10	0.09
Specific Activity Scale score (per 1 MET)	0.90	0.80–1.03	0.12
Progressive AS	2.47	1.38–4.39	0.002
Diabetes	1.40	0.86–2.31	0.18
Ischemic heart disease	2.50	1.55–4.03	< 0.001
Heart failure	1.54	0.94–2.51	0.08
Peripheral artery disease	0.89	0.53–1.49	0.66

Other abbreviations as in Table 1

*CI* confidence interval

Univariate Cox proportional hazard analysis demonstrated that independent predictors of all-cause mortality were age, SAS score, progressive AS, history of ischemic heart disease, PAD, serum albumin level, and C-reactive protein level. Multivariate Cox proportional hazard analysis showed that age, SAS score, and serum albumin and C-reactive protein levels were independent predictors for all-cause mortality (Table 4).

**Table 4:** Multivariate Cox proportional hazard analysis regarding prediction of all-cause mortality

Variable	Adjusted hazard ratio	95% Cl	*P*
Age (per 1 year)	1.03	1.01–1.05	0.009
Specific Activity Scale score (per 1 MET)	0.77	0.66–0.89	< 0.001
Progressive AS	1.57	0.89–2.77	0.12
Ischemic heart disease	1.54	0.95–2.48	0.077
Peripheral artery disease	1.10	0.69–1.77	0.68
Serum albumin level (per 1 g/dL)	0.54	0.31–0.95	0.032
C-reactive protein (per 1 mg/dL)	1.10	1.03–1.19	0.007

Abbreviations as in Tables 1 and 3

Univariate Cox proportional hazard analysis regarding prediction of cardiac death revealed that SAS score, presence of progressive AS, history of diabetes, history of ischemic heart disease, and serum levels of albumin and C-reactive protein were significant independent variables. Stepwise multivariate Cox proportional hazard analysis found that SAS score, progressive AS, and history of ischemic heart disease were independent predictors for cardiac death (Table 5).

**Table 5:** Stepwise multivariate Cox proportional hazard analysis regarding prediction of cardiac death

Variable	Adjusted hazard ratio	95% Cl	*P*
Specific Activity Scale score (per 1 MET)	0.70	0.58–0.85	< 0.001
Progressive AS	4.21	2.10–8.46	< 0.001
Ischemic heart disease	3.04	1.59–5.83	< 0.001

Abbreviations as in Tables 1 and 3

## Discussion

In this prospective cohort study, we compared the prognosis of maintenance hemodialysis patients with progressive AS with the prognosis of those without AS with a median follow-up period of 4.1 years. The present study has two main findings. First, the rates of cardiac events, all-cause mortality, and cardiac death were higher in patients with progressive AS than in those without AS. Second, the presence of progressive AS and history of ischemic heart disease independently predicted cardiac events and cardiac death. In contrast, progressive AS was not predictive of all-cause mortality.

Our study indicated that the cardiac event rate was higher in hemodialysis patients with progressive AS than in those without AS. Especially, the cumulative rate of cardiac death was more than three times higher in patients with progressive AS than in those without AS. Furthermore, progressive AS—together with a history of ischemic heart disease—was predictive of cardiac events and cardiac death in those patients. Although the mean left ventricular ejection fraction was comparable between patients with or without progressive AS, *E*/*e*′ was significantly higher in those with progressive AS. Hence, diastolic dysfunction resulting from myocardial extracellular expansion and replacement fibrosis due to AS may cause an imbalance in myocardial oxygen supply and demand and thus influence the onset of cardiac events, including cardiac death [16]. Careful cardiovascular management and body fluid control are needed for patients with progressive AS to prevent cardiac events.

Our analysis revealed that the 3-year rates of cardiac events and cardiac mortality in hemodialysis patients with progressive AS were 44.9% and 26.4%, respectively. Some studies have reported the risk of cardiac events in patients with progressive AS in the general population. For example, the cumulative incidence of heart failure hospitalization and aortic valve replacement at 3 years of follow-up were 47% in patients with moderate AS and reduced left ventricular ejection fraction [6]. In addition, the composite of ischemic cardiovascular events and aortic valve-related events occurred in 39% of asymptomatic patients with progressive AS during a median follow-up of 4.3 years [3]. In a large cohort study, the 5-year rate of cardiovascular mortality was 31.6% in the patients with progressive AS [7]. Given these previous reports, the rates of cardiac events and cardiac mortality in hemodialysis patients with progressive AS may be comparable to those in the general population with progressive AS.

In our study, the 3-year all-cause mortality in hemodialysis patients with progressive AS was 40.5%. This mortality may be higher than that of subjects with progressive AS in the general population (18–28%) [6–8]. The present study found that the increased incidence of all-cause mortality in patients with progressive AS was not associated with a diseased aortic valve but instead was related to their comorbidities. Previous reports that assessed the general population obtained conflicting results regarding the involvement of progressive AS in all-cause death. Aortic *V*_max_ was an independent predictor of the composite of death and cardiac events in patients with moderate AS and a reduced left ventricular ejection fraction [6]. In a large cohort study, multivariate Cox proportional hazard analysis demonstrated that mean ∆*P* ≥ 20 mmHg was an independent predictor for all-cause mortality; however, this analysis was adjusted only for age and gender and disregarded other clinical risk factors [7]. Another group found that age and the presence of comorbidities predicted all-cause mortality in patients with moderate AS, but moderate AS itself did not predict it [8]. The results of that study were similar to ours, in which age, low physical activity, high C-reactive protein level, and hypoalbuminemia—all of which are known predictors for mortality in hemodialysis patients—predicted all-cause mortality in our patients [8, 17–19]. We consider that hemodialysis patients with progressive AS should be aggressively managed for these risk factors because they could adversely affect the outcomes of future aortic valve interventions.

In our present study, the prevalence of exercise-related symptoms was comparable between patients with and without progressive AS. Patients receiving hemodialysis often have these symptoms, probably because of excess body fluid, heart failure, or ischemic heart disease [3]. Therefore, discerning whether AS is a direct cause of these symptoms may be difficult.

The main limitation of this research was that it was a single-center study. In addition, the time interval between echocardiography and subsequent hemodialysis differed for each patient. The timing of hemodialysis might have variably influenced the cardiac hemodynamic condition.

## Conclusions

In conclusion, compared with those without AS, hemodialysis patients with progressive AS had higher rates of cardiac events, cardiac death, and mortality. In addition, progressive AS was predictive of cardiac events and cardiac death in hemodialysis patients. The higher all-cause mortality in those with progressive AS was related to their comorbidities.

## Data Availability

The datasets generated and/or analyzed during this study are available from the corresponding author on reasonable request.

## Abbreviations

Aortic *V*_max_: Peak aortic jet velocity
AS: Aortic stenosis
AVA: Aortic valve area
*E*/*e*′: The ratio between early mitral inflow velocity and mitral annular early diastolic velocity
Mean ∆*P*: Mean pressure gradient across the aortic valve
METs: Metabolic equivalents
PAD: Peripheral artery disease
SAS: Specific Activity Scale
SVI: Stroke volume index
